# Neuroimaging features of immune-related adverse events due to immune checkpoint inhibitor therapy

**DOI:** 10.1186/s13244-025-01999-3

**Published:** 2025-06-12

**Authors:** Arian Lasocki, Lavinia Spain

**Affiliations:** 1 Department of Cancer Imaging, Peter MacCallum Cancer Centre, Melbourne, Victoria Australia; 2 Sir Peter MacCallum Department of Oncology, The University of Melbourne, Parkville, Victoria Australia; 3 Department of Radiology, The University of Melbourne, Parkville, Victoria Australia; 4 Department of Medical Oncology, Peter MacCallum Cancer Centre, Melbourne, Victoria Australia

**Keywords:** Immune checkpoint inhibitors, Immune-related adverse events, Meningoencephalitis, Vasculitis, Magnetic resonance imaging

## Abstract

**Abstract:**

Immune checkpoint inhibitors, a type of intravenous immunotherapy targeting T cells, are being increasingly used in cancer treatment. They work by increasing the immune system’s response to tumour cells, through blockade of inhibitory “checkpoint” receptors. Immune checkpoint inhibitors commonly induce immune-related adverse events (irAEs) affecting multiple organ systems. Hypophysitis is strictly an endocrine irAE, but is the most common irAE identified on neuroimaging. True neurologic irAEs are rare and widely varied. Examples include meningitis, encephalitis, vasculitis, demyelinating syndromes and neuritis. Some neurologic irAEs are not associated with neuroimaging findings (for example, neuromuscular junction disorders), while in others, imaging findings are present in only a proportion of patients (for example, encephalitis). Diagnosing, or at least considering, a neurologic irAE is important for instigating the appropriate management and optimising patient outcomes. This educational review illustrates irAEs that may be identified on neuroimaging and provides practical tips for optimising diagnosis, including relevant clinical considerations.

**Critical relevance statement:**

Immune checkpoint inhibitors, which are being increasingly used in cancer treatment, commonly induce immune-related adverse events. This educational review illustrates the range of immune-related adverse events for which neuroimaging plays a key role in diagnosis.

**Key Points:**

- Immune checkpoint inhibitors commonly result in immune-related adverse events (irAEs) affecting multiple organ systems.
- Hypophysitis, the most common irAE identified on neuroimaging, is characterised by transient pituitary enlargement.
- True neurologic irAEs are rare and include meningitis, encephalitis, vasculitis, demyelination and neuritis.
- An understanding of the overall clinical picture is important for supporting the diagnosis.

**Graphical Abstract:**

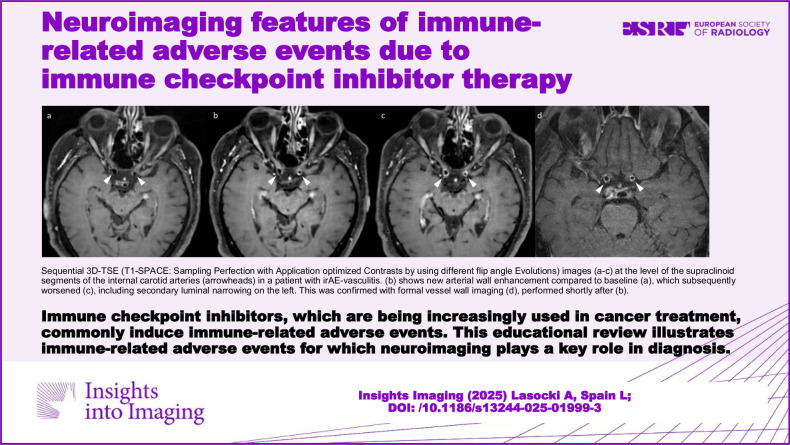

## Introduction

Immune checkpoint inhibitors (ICIs), a type of intravenous immunotherapy targeting T cells, are being increasingly used in cancer treatment. Their use is expanding to a broader range of cancers, including melanoma, lung and urologic cancers, and to earlier stages of disease, such as the neoadjuvant and adjuvant realms [1]. ICIs work by increasing the immune system’s response to tumour cells through blockade of inhibitory “checkpoint” receptors. Molecular targets and the agents which target them include cytotoxic T-lymphocyte associated protein 4 (CTLA-4; targeted by ipilimumab), programmed cell death protein 1 (PD-1; agents include nivolumab, pembrolizumab and cemiplimab), programmed cell death ligand 1 (PD-L1; for example, atezolizumab, avelumab and durvalumab) and lymphocyte activation gene 3 (LAG-3; including relatlimab) [2, 3]. Agents targeting different immune checkpoint pathways can be combined to increase efficacy; for example, combination therapy with ipilimumab and nivolumab has become a standard-of-care in metastatic melanoma. ICIs can also be combined with other anti-cancer agents, such as cytotoxic chemotherapy and targeted therapies (such as anti-VEGFR tyrosine kinase inhibitors) [2].

Increased immune activity through the use of ICIs strengthens the body’s own ability to recognise and fight the cancer, but also commonly results in immune-related adverse events (irAEs) affecting multiple different organ systems. Overall, irAEs occur in the majority of patients treated with ICIs, and high-grade ICIs occur in 10–50%, with combination ICI approaches yielding the highest rates [1, 4]. The most common irAEs are dermatologic, cardiac, endocrine, gastrointestinal, pulmonary and renal [1]. Hypophysitis is strictly an endocrine irAE but is the most common irAE identified on neuroimaging. True neurologic irAEs are less common, with a described incidence of 4–6% for monotherapy and 12% for combination anti-CTLA4 and anti-PD-1 [5]. They can vary widely in their presentation and do not necessarily mimic idiopathic autoimmune conditions. Broad groups include meningitis, encephalitis, demyelinating syndromes, vasculitis, neuropathy, neuromuscular junction disorders and myopathy [6], though this is not an all-inclusive list. Some neurologic irAEs are not associated with neuroimaging findings (for example, myasthenia gravis or Lambert-Eaton syndrome), while in others, imaging findings are present in only a proportion of patients (for example, encephalitis).

Most irAEs occur within 12 weeks of commencing ICIs, but they can occur months or even years after treatment [7]. They generally occur earlier with combination therapy than monotherapy [8]. In one series, neurologic irAEs were reported as occurring a median of 6 weeks after commencing treatment [5]. Given their rarity, it is difficult to distinguish between individual neurologic irAE syndromes. Hypophysitis tends to occur slightly later, usually about 2–3 months after commencing treatment [9–11]. Cases associated with PD-1 inhibitors generally develop later compared to patients treated with CTLA-4 inhibitors (whether single-agent or in combination) [10, 11]. In general, irAEs are more common with CTLA-4 inhibitors than PD-1 or PD-L1 inhibitors [12], and the incidence increases further if using combination therapy [8]. Of note, hypophysitis has a particular association with ipilimumab [9].

Identification of irAEs is important for several reasons. The presentation of irAEs is often nonspecific and therefore may overlap clinically with progression of the patient’s metastatic disease or other differential diagnoses, such as infection. The identification of an irAE is thus important to exclude a more sinister cause for the patient’s presentation. Diagnosing, or at least suggesting, an irAE is important for guiding appropriate management. Neurologic irAEs have a relatively high mortality [13], and prompt diagnosis is important for improving outcomes [14]. Treatment of neurologic irAEs generally comprises withholding ICIs and empiric corticosteroids, which leads to improvement in many cases [14]. Additional options for neurologic irAEs refractory to these initial steps include plasmapheresis, intravenous immunoglobulin and other immunosuppressive agents such as rituximab, cyclophosphamide and infliximab [14]. There may also be longer-term implications for the patient. For example, hypophysitis will often lead to hypopituitarism, requiring long-term hormone replacement [9], and subacute and chronic toxicity may develop in more than 40% of patients impacted by neurologic irAEs [15]. Even when an intracranial finding is convincingly not attributable to progression of metastatic disease, it is important to determine whether or not it is likely due to ICIs or incidental, as the latter will allow for the continuation of ICIs, which is important for optimising the treatment of the patient’s cancer.

This educational review illustrates irAEs that may be identified on neuroimaging and provides practical tips for optimising diagnosis.

### Hypophysitis

**Fig. 1:**
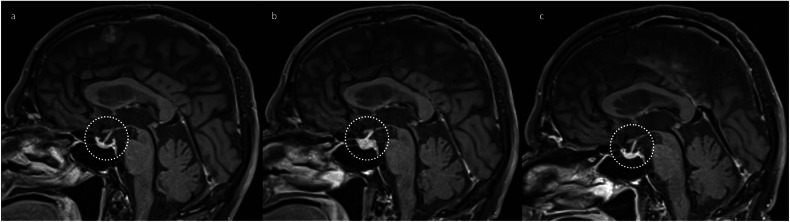
Serial sagittal post-contrast T1-weighted images of the pituitary gland (dotted circle), demonstrating the typical temporal evolution of hypophysitis (which developed after two cycles of combination ipilimumab and nivolumab for metastatic melanoma): enlargement (**b**) compared to baseline (**a**), followed by a decrease in size (**c**)

**Fig. 2:**
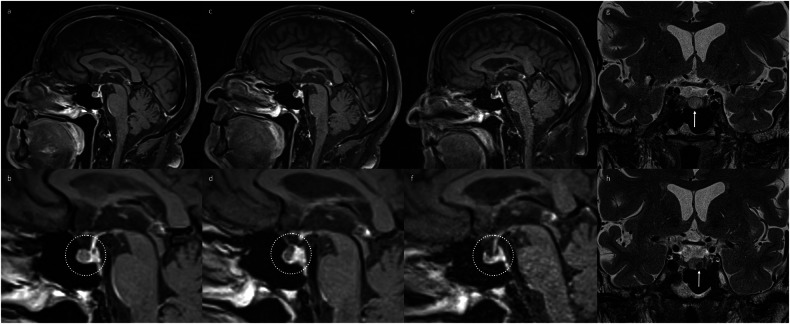
Serial sagittal post T1-weighted images of the pituitary gland (top row) and corresponding zoomed panels (bottom row, dotted circles) in a patient who had received four cycles of combination ipilimumab and nivolumab for metastatic melanoma, demonstrating an atypical appearance of hypophysitis, related to the presence of a pre-existing Rathke cleft, visible as a round non-enhancing area at the anterior aspect of the pituitary gland prior to ICI therapy (**a**, **b**). Despite the Rathke cleft cyst, enlargement of the enhancing pituitary tissue is visible (**c**, **d**) and leads to a change in the configuration of the cyst. Later (**e**, **f**), both the cyst and the pituitary tissue itself appear smaller than at baseline. The findings of hypophysitis occurred while on low-dose oral corticosteroids for a treatment-induced rash and resolved without additional treatment. Dedicated coronal T2-weighted images obtained at baseline further characterise the anterior Rathke cleft cyst, showing it to be predominantly T2-hyperintense (**g**), except for a T2-hypointense intracystic nodule (**h**), consistent with a Rathke cleft cyst [30]

Hypophysitis is the most common irAE visualised on neuroimaging. Hypophysitis manifests as transient enlargement of the pituitary gland, which is usually mild (Fig. 1). In most cases, the pituitary size is only just about the above limit of normal or even within the normal range [16]. This makes it important to correlate with pre-treatment neuroimaging, especially if the pituitary gland measures towards the upper limit of normal or larger (e.g., ≥ 7 mm craniocaudally) [17]. The enlargement resolves within a few months, and the pituitary gland may subsequently become smaller than on pre-treatment baseline imaging (Fig. 2). In most cases, the enhancement of the pituitary gland remains homogeneous. Heterogeneous enhancement can occur [16] and does not specifically suggest an alternate diagnosis if the expected temporal evolution is demonstrated. Mild, smooth thickening of the pituitary stalk is commonly observed [16]. Being an acute process, the sella should be of normal size (i.e., not expanded). In contrast, features which suggest an alternate diagnosis include pituitary size > 2 cm, the visualisation of a distinct lesion within the pituitary tissue, sellar expansion, extension into adjacent structures such as the cavernous sinus, and nodular thickening of the pituitary stalk [16, 17].

Not uncommonly, hypophysitis is visualised on routine re-staging imaging without a suggestive clinical history [18]. In some cases, this is because the imaging was requested before symptoms developed, but occasionally, imaging evidence of hypophysitis truly precedes the development of symptoms. In most cases, hypophysitis can be readily diagnosed on a standard imaging protocol used for intracranial disease surveillance, though a dedicated pituitary protocol can be helpful if there is diagnostic uncertainty, especially if pre-treatment baseline imaging is not available. Correlation with 18F-fluorodeoxyglucose positron-emission tomography is also helpful if performed; hypophysitis will result in tracer uptake [19], which should not normally be present in the sella turcica.

### Focal encephalitis

A variety of clinical and imaging patterns of irAE-meningoencephalitis have been described, though neuroimaging findings are not always present. Farina et al divided irAE-meningoencephalitis into two groups based on the clinical presentation—focal encephalitis and non-focal meningoencephalitis [20]. The most common focal encephalitis in their series was limbic encephalitis, though noting that about two-thirds of patients in this series had lung cancer, of whom approximately half had small cell lung cancer [20]. Other focal encephalitis syndromes that can be observed include cerebellitis, rhombencephalitis, opsoclonus-myoclonus-ataxia syndrome and stiff-person syndrome [6].

**Fig. 3:**
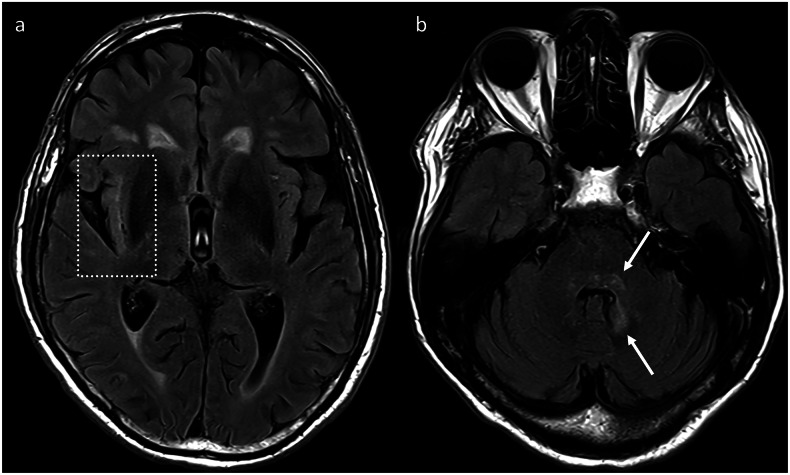
Axial FLAIR images in a patient with irAE-encephalitis demonstrating high signal in the right insula (**a**, dotted square), as well as in the dorsal pons extending to the adjacent left cerebellar hemisphere (**b**, arrows). The patient had initially presented with progressive unsteadiness after 4 cycles of cemiplimab for metastatic cutaneous squamous cell carcinoma. CSF analysis demonstrated a lymphocytosis, without any neuronal antibodies

Abnormalities were demonstrated on MRI in over half (62%) of patients with a focal irAE-encephalitis in the aforementioned series by Farina et al [20]. MRI may demonstrate new areas of FLAIR hyperintensity, for example, affecting the limbic system or posterior fossa (Fig. 3). The use of volumetric FLAIR can aid in the detection of such parenchymal FLAIR hyperintensities. The neuroimaging findings often overlap with meningoencephalitis, however. For example, abnormal meningeal enhancement can be seen in association with a clinical picture of focal encephalitis [20]. Overlap between the different meningoencephalitis syndromes is also common, and a concomitant peripheral neuropathy or myelitis can also be observed [20].

### Non-focal meningoencephalitis

**Fig. 4:**
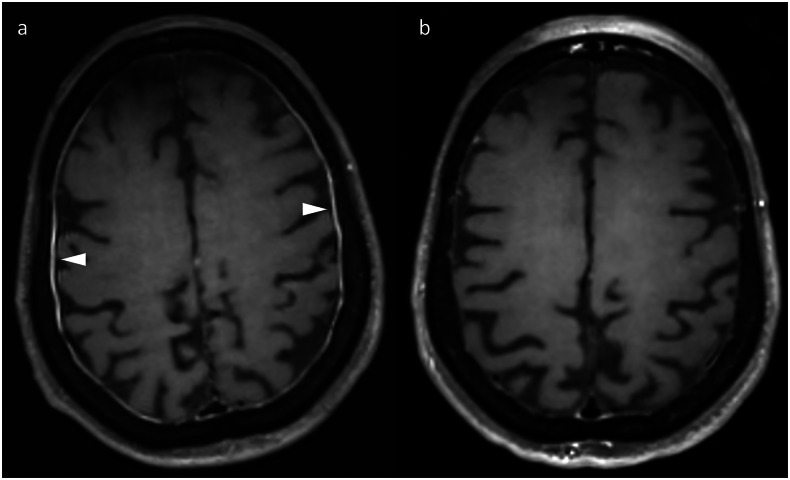
Serial axial post-contrast T1-weighted images in a patient who developed clinical features of irAE-meningoencephalitis while on maintenance nivolumab for metastatic melanoma. The images show abnormal pachymeningeal enhancement (**a**, arrowheads), which subsequently resolved after treatment (**b**)

**Fig. 5:**
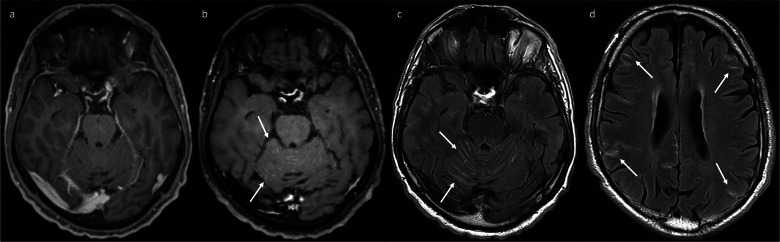
Different post-contrast imaging sequences in a patient with clinical features of irAE-encephalitis after cemiplimab for metastatic cutaneous squamous cell carcinoma, demonstrating the value of using different post-contrast sequences. Sulcal enhancement (arrows) visible on 3D-TSE (**b**) is barely visible on 3D-GRE (**a**). It is arguably best appreciated on the post-contrast FLAIR sequence (**c**), which also demonstrated enhancement within several cerebral sulci further superiorly (**d**)

In the above series by Farina et al, non-focal meningoencephalitis was slightly more common than focal encephalitis in patients without lung cancer, and the vast majority of patients with meningoencephalitis had a non-lung primary [20]. This indicates that tumour histology influences the relative incidence of different irAEs, as has also been observed outside the brain—for example, vitiligo is most common in patients treated for metastatic melanoma [12]. It is more common for neuroimaging to be negative in patients with a non-focal meningoencephalitis than in focal encephalitides, which was the case in 67% of the non-focal meningoencephalitis cases in the above series [20]. Nevertheless, MRI may demonstrate abnormal meningeal enhancement, which can be leptomeningeal or pachymeningeal (Fig. 4); the former in particular can mimic leptomeningeal metastatic disease (LMD). Similar to the overlapping MRI features discussed in the context of encephalitis, parenchymal changes can also be seen in patients with a more general meningoencephalitis presentation [20]. In our experience, volumetric turbo spin echo (3D-TSE) post-contrast T1-weighted imaging better demonstrates abnormal meningeal enhancement than gradient echo-based equivalents (3D-GRE), and post-contrast FLAIR is also a useful adjunct (Fig. 5). Cerebrospinal fluid (CSF) analysis is always suggested in conjunction with imaging in cases of suspected immune-related meningoencephalitis and may help exclude LMD. It should be performed after MRI to avoid confounding the MRI appearances.

### Demyelination

**Fig. 6:**
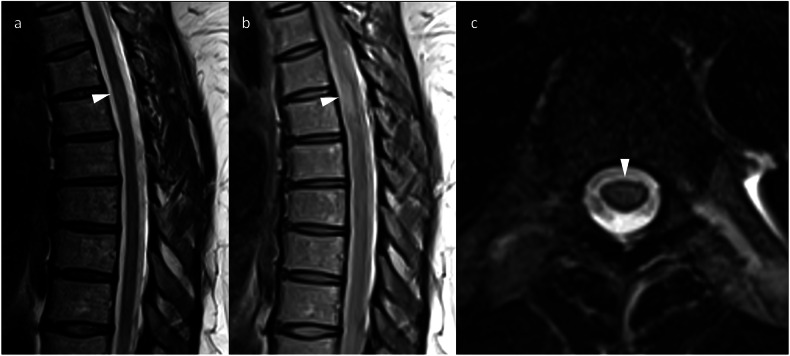
Sagittal T2 (**a**) and PD (**b**) images in a patient on maintenance nivolumab after induction with combination ipilimumab and nivolumab for metastatic melanoma show an area of high signal in the thoracic spinal cord (arrowheads), confirmed in the axial plane (**c**), consistent with irAE-demyelination

Pre-existing autoimmune conditions make the delivery of ICIs more complicated, as ICI may result in a disease flare. ICIs can also cause demyelination in patients without a prior history of a disease such as multiple sclerosis. The MRI appearances of demyelination after ICI therapy are equivalent to demyelination occurring outside of this context (Fig. 6). Optic neuritis can also occur [21].

### Neuritis

**Fig. 7:**
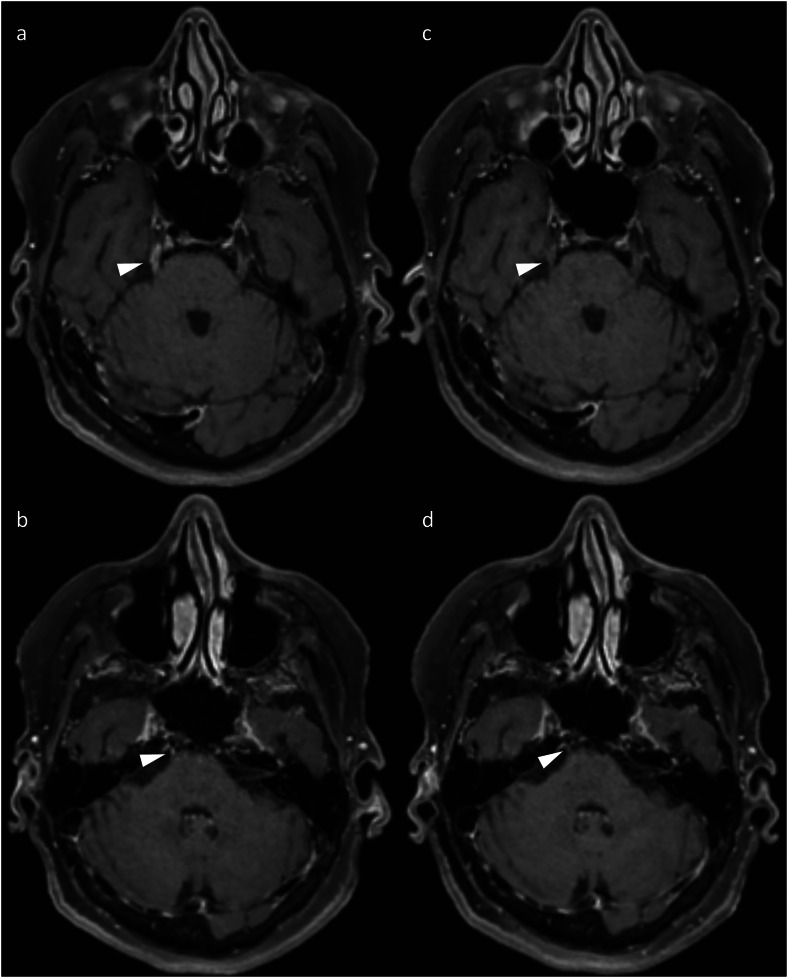
Axial post-contrast T1-weighted images of a patient who developed diplopia on maintenance nivolumab after induction with combination ipilimumab and nivolumab for metastatic melanoma, demonstrating evidence of cranial neuritis (arrowheads), characterised by the development of enhancement of the right trigeminal nerve (**a**) and right abducens nerve (**b**). The abnormal enhancement subsequently resolved (**c**, **d**) after withholding ICI therapy (without the addition of corticosteroids), together with the resolution of the patient’s diplopia

Similar to irAE-meningitis, neuritis is particularly challenging to diagnose due to the overlap with LMD. This manifests on imaging as enhancement of multiple spinal nerve roots (polyradiculitis) and/or cranial nerves [6, 22, 23] (Fig. 7). Importantly, the nerve enhancement should be smooth [6]; a more nodular appearance would instead suggest LMD.

### Cerebral vasculitis

**Fig. 8:**
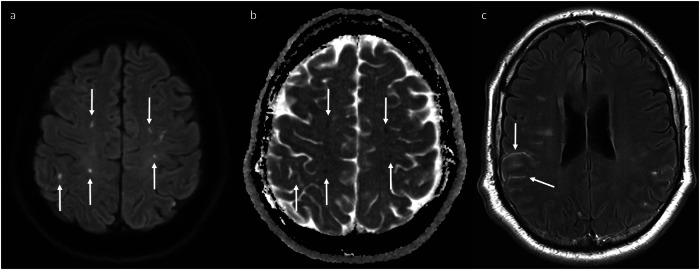
This patient developed evidence of irAE vasculitis while on maintenance nivolumab for unresectable stage III melanoma. Diffusion-weighting imaging (**a**) and apparent diffusion coefficient map (**b**) show foci in both cerebral hemispheres exhibiting high and low signal (arrows), respectively, consistent with recent infarcts in multiple vascular territories. The patient also had abnormal sulcal leptomeningeal enhancement on the post-contrast FLAIR sequence (**c**, arrows). The leptomeningeal enhancement subsequently resolved after treatment with intravenous methylprednisolone and cyclophosphamide, confirming that it was related to the irAE-vasculitis rather than leptomeningeal metastatic disease

**Fig. 9:**
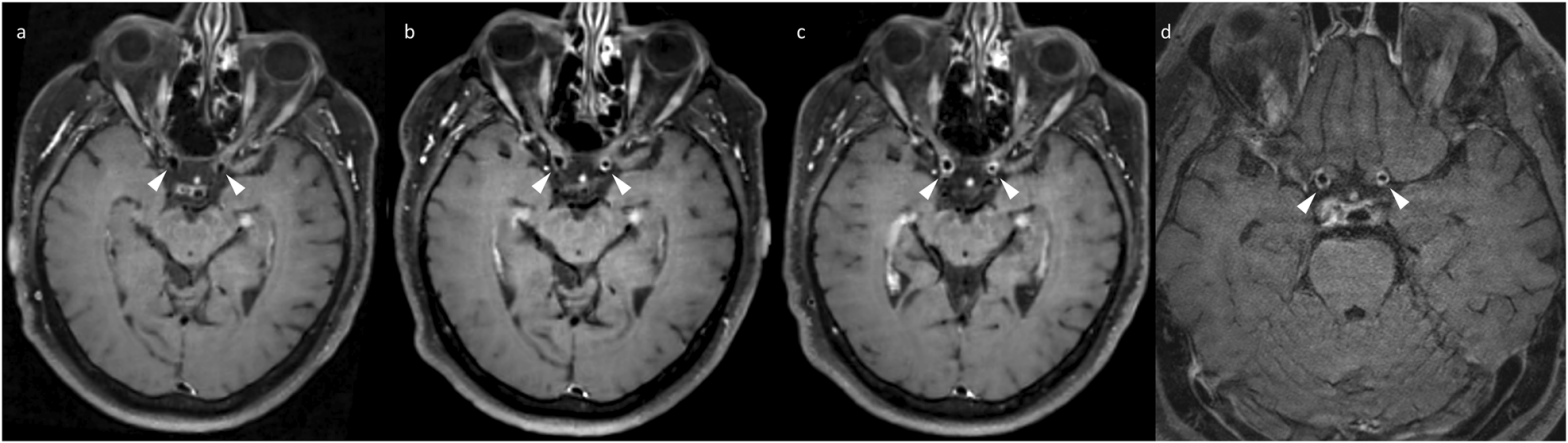
Sequential 3D-TSE images (**a**–**c**) at the level of the supraclinoid segments of the internal carotid arteries (arrowheads) in a patient who developed irAE-vasculitis while on maintenance nivolumab for melanoma. **b** Shows new arterial wall enhancement compared to baseline (**a**), which subsequently worsened (**c**), including secondary luminal narrowing on the left. This was confirmed with formal vessel wall imaging (**d**), performed shortly after (**b**)

Cerebral vasculitis should be considered in patients being treated with ICIs who have evidence of ischaemia, especially if involving multiple vascular territories (Fig. 8), though infarcts unrelated to ICIs therapy can also occur. Abnormal leptomeningeal enhancement may also be observed (see Fig. 8); thus, there is an overlap with the appearances of meningoencephalitis. 3D-TSE post-contrast imaging is being increasingly preferred over 3D-GRE due to an increased sensitivity for the detection of intracranial metastases [24–27]. Another benefit of 3D-TSE in this context is that it may allow some assessment of arterial wall enhancement (Fig. 9), as signal within the vessel lumen is generally suppressed; in contrast, homogeneous vascular enhancement with 3D-GRE prevents distinction between the arterial lumen and any potential mural enhancement. Formal vessel wall imaging can also be used to increase diagnostic confidence [6] (see Fig. 9).

### Other inflammatory conditions

**Fig. 10:**
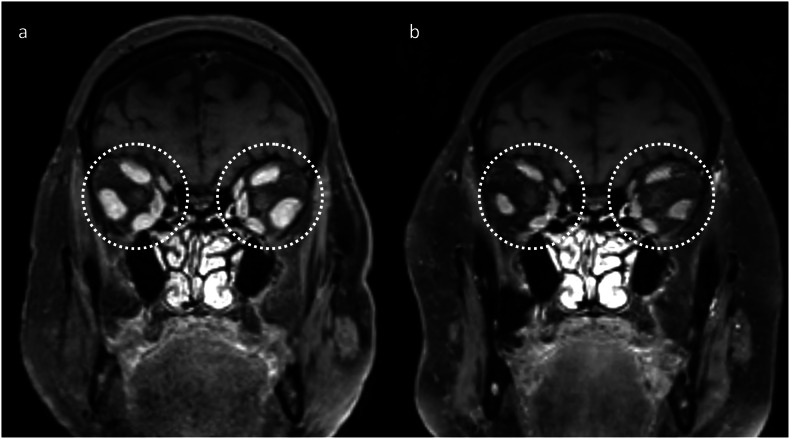
Post-contrast T1-weighted imaging in the coronal plane in a patient who had recently commenced pembrolizumab for a urothelial carcinoma. This demonstrates enlargement of the extraocular muscles (**a**, dotted circles), which resolved after treatment with corticosteroids (**b**), consistent with orbital myositis

**Fig. 11:**
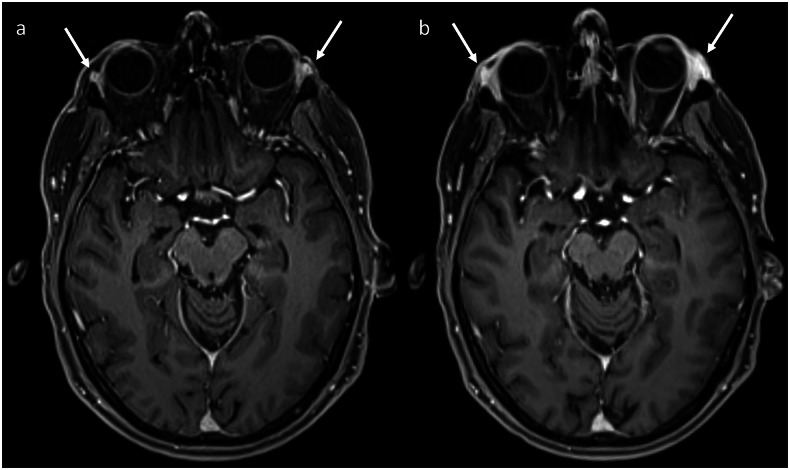
Axial post-contrast T1-weighted images before (**a**) and after (**b**) commencing ICI therapy (combination ipilimumab and nivolumab followed by maintenance nivolumab) in a patient with metastatic melanoma who developed eye pain after ICIs, demonstrating enlargement of the lacrimal glands, consistent with dacryoadenitis

ICIs have also been reported to cause or potentiate rarer immune-related neurologic conditions outside of the main groups described above, such as cerebral amyloid angiopathy-related inflammation [28]. Additionally, ICIs may cause inflammatory enlargement of adjacent structures, such as the extraocular muscles (orbital myositis; Fig. 10) or lacrimal glands [21] (dacryoadenitis; Fig. 11).

### Clinical features

**Fig. 12:**
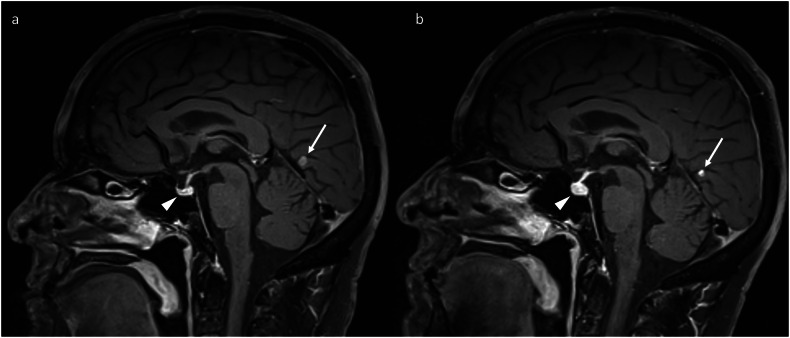
Sagittal post-contrast T1-weighted images before (**a**) and after (**b**) two cycles of combination ipilimumab and nivolumab for metastatic melanoma demonstrate enlargement of the pituitary gland (arrowheads) typical of hypophysitis, together with a decrease in the size of the occipital metastasis (arrows)

Due to overlap in the imaging features of neurologic irAEs with other key differentials, it is often difficult to make a definitive diagnosis of a neurologic irAE at the time of initial imaging. irAEs may not present in the same manner as idiopathic autoimmune diseases, and onset can be either subacute or acute. An understanding of the overall clinical picture is important for supporting the diagnosis. One helpful clue to a neurologic irAE is a discordant clinical picture, with new symptoms or imaging findings occurring despite evidence of a response to ICI therapy (Fig. 12). A history of other irAEs is also important, as it is common for patients with rare irAEs to have also experienced other, more common irAEs, either previously or concurrently. CSF analysis is often recommended, for example, in the case of a meningoencephalitis or radiculitis, both to provide support for an inflammatory process (including potentially the detection or relevant auto-antibodies) and to exclude other possibilities such as leptomeningeal metastatic disease or infection [6]. Other tests, such as an electroencephalogram, electromyography or nerve conduction studies, can also be helpful depending on the specific irAE [29]. Not infrequently, making a definitive diagnosis requires demonstration of improvement with immunosuppressive treatment; thus, imaging follow-up is important for patients with positive imaging findings.

## Conclusion

Neurologic irAEs are rare and varied. The diagnosis may be challenging, as the clinical and imaging features often overlap with other differentials such as metastatic deposits, including leptomeningeal metastatic disease or infection. Diagnosing, or at least considering, a neurologic irAE is important for instigating the appropriate management and optimising patient outcomes.

## Abbreviations

3D-GRE: Volumetric gradient recalled echo
3D-TSE: Volumetric turbo spin echo
CSF: Cerebrospinal fluid
CTLA-4: Cytotoxic T-lymphocyte associated protein 4
ICI: Immune checkpoint inhibitor
irAE: Immune-related adverse event
LMD: Leptomeningeal metastatic disease
PD-1: Programmed cell death protein 1
PD-L1: Programmed cell death ligand 1
